# The impact of artificial intelligence application on employees' job insecurity: the moderating roles of self-efficacy and transformational leadership

**DOI:** 10.3389/fpsyg.2026.1786588

**Published:** 2026-06-03

**Authors:** Wenxiu Fu, Hui Zhang

**Affiliations:** 1 Shandong Management University, School of Business Administration, Jinan, China; 2 Shandong University of Finance and Economics, School of Business Administration, Jinan, China

**Keywords:** artificial intelligence application, conservation of resources theory (COR), employees' job insecurity, self-efficacy, transformational leadership

## Abstract

**Introduction:**

Against the backdrop of the intelligent era, the widespread application of artificial intelligence (AI) has fundamentally reshaped the internal and external developmental ecosystems of organizations, exerting a profound impact on employees' work-related psychological states. Drawing on the Conservation of Resources Theory and the Cognitive Appraisal Theory of Stress, this study empirically explores the underlying mechanism through which AI application influences employees' job insecurity.

**Methods:**

Data for this study were collected using a mixed online and offline distribution method, with all measures administered through employee self-reported questionnaires. A total of 453 questionnaires were distributed, including 242 online and 211 offline. We received 449 questionnaires, with 242 from the online channel and 207 offline. Following a stringent validity screening process conducted by the research team, 411 valid questionnaires were retained for analysis (219 online and 192 offline), resulting in an effective response rate of 90.73%.

**Results:**

There is a significant U-shaped relationship between AI application and employees' job insecurity: moderate AI application reduces insecurity, whereas excessive application heightens it. Self-efficacy negatively moderates this relationship by strengthening the insecurity-reducing effect of moderate AI application and weakening the insecurity-enhancing effect of excessive application. Transformational leadership similarly exerts a negative moderating effect, suggesting that both individual psychological resources and supportive leadership can buffer employees' insecurity responses to varying levels of AI application in digitality transforming organizational contexts more effectively.

**Discussion:**

This study advances research on AI-enabled workplace changes by revealing a U-shaped effect of AI application intensity on employees' job insecurity. It explains this relationship through the dual mechanisms of resource gain and resource threat. It further incorporates self-efficacy and transformational leadership as boundary conditions, thereby clarifying when AI application alleviates or intensifies employees' job insecurity. These findings enrich the theoretical understanding of employees' job insecurity within the context of AI application, and offer empirical insights for managing employee wellbeing and refining human resource strategies during organizational digital transformation.

## 1 Introduction

The breakthrough advancement of digital-intelligent technology and information technology has ushered in the full arrival of the Industry 4.0 era. As a foundational general-purpose technology, artificial intelligence (AI) is giving rise to a new round of technological revolution and industrial transformation. In contemporary China, AI application within enterprises has grown increasingly extensive and in-depth, spanning intelligent production processes to management decision support, and profoundly reshaping organizational operation paradigms and market competition landscapes (Malloy and Gonzalez, 2024; Raisch and Fomina, 2025).

However, while the AI application delivers efficiency gains and innovation opportunities, it also gives rise to a host of challenges, including rapidly evolving skill requirements, blurred job roles, and anxiety over career development among employees (Raisch and Krakowski, 2021). Researchers acknowledge that AI is reshaping the internal and external environments of enterprise operations. Externally, AI application has profoundly transformed industrial competitive landscapes and business models, intensifying market competition (Chan and Choi, 2025; Ledro et al., 2025). Internally, AI application has boosted production efficiency, transformed work practices, optimized customer experiences, and accelerated organizational innovation and transformation (Li et al., 2022). Notably, the substitution effect of AI on certain types of labor has exacerbated employees' concerns over unemployment risk and skill depreciation, substantially elevating their job insecurity and thus negatively impacting their work experiences (Yam et al., 2023). Such technological adoption not only reshapes the organizational development ecosystem but also fundamentally transforms employees' working patterns, performance dynamics, and job content (Zhou et al., 2025). Therefore, against the backdrop of rapid AI technological iteration, exploring the impact of AI application on employees' sense of job insecurity holds significant theoretical value and practical implications for organizations to formulate evidence-based and effective human resource management strategies.

Employees' job insecurity refers to the perception of an individual that their job continuity or core job characteristics are under threat, which constitutes a stressful psychological experience (Jiang et al., 2022). The Conservation of Resources Theory posits that individuals have an inherent motivation to acquire, maintain, and protect their important resources. The work itself, as well as occupational resources such as skill value, work ability, and career stability, all fall within the category of core resources that individuals prioritize in safeguarding. When individuals perceive that such resources may be lost, they are prone to experience psychological stress. The formation of employees' job insecurity is influenced by multiple intertwined factors, including macroeconomic fluctuations, organizational changes, technological advancements, and so on (Hsieh and Kao, 2022; Qian et al., 2022). During the rapid development of digital and intelligent technologies, AI application is fundamentally reshaping the working environment of employees, presenting a two-way influence characteristic. On the one hand, AI drives the iterative improvement of skills, accelerates the update of knowledge, optimizes work processes, and enhances the professional competence of employees, thereby effectively improving their work efficiency and performance (Li et al., 2024; Yang et al., 2025). On the other hand, AI application directly impacts the key career resources that employees possess, redefines the value of positions, and undermines their work enthusiasm. At the same time, it also intensifies employees' concerns about job continuity and future career stability (Peltokorpi and Allen, 2024; Shoss and Vancouver, 2024).

Against the complex interplay between AI application and employees' individual perceptions and behaviors, employees' personal psychological resources and organizational contextual factors both serve as critical boundary conditions. According to the Cognitive Appraisal Theory of Stress, an individual's assessment of the stressor determines the subsequent psychological and behavioral response patterns. Self-efficacy, as the core individual psychological resource, is an individuals' belief in their ability to complete a specific task (Jeong and Jeong, 2025). When employees exhibit a high level of self-efficacy, they will perceive the insecurity arising from AI application as a challenging stressor and thus develop a proactive willingness to engage in self-renewal and capability enhancement. This positive cognitive assessment may change the effect of AI application on employees' job insecurity (Alessandri et al., 2025; Kim and Lee, 2025). Leadership behavior, as an important organizational contextual factor, can build employees' psychological security and enrich their resources for coping with stress. Transformational leadership can enhance employees' sense of organizational belonging and environmental adaptability by outlining inspiring visions, providing personalized care, and stimulating employees' intellectual engagement (Liu and Nie, 2024). During the application of AI, the enabling support and scientific guidance provided by transformational leadership can effectively reduce employees' perception of environmental uncertainty. It can also strengthen their confidence in mastering new technologies, and thereby mitigate the negative emotional responses that may be elicited by AI application (Yuan, 2025; Zainab et al., 2022).

In conclusion, existing studies have deepened our understanding of the mechanism underlying the relationship between AI application and employees' job insecurity, providing a theoretical foundation for examining the effect of the former on the latter. Nevertheless, two core issues remain underexplored and warrant further investigation. On the one hand, although some scholars have examined the impact of AI application on employees' work-related experiences, findings remain inconsistent. Accordingly, a critical question arises, that is, what specific effect does AI application exert on employees' job insecurity? Whether AI application can effectively reduce employees' job insecurity through its innovative effect or intensify this psychological experience via its substitution effect remains to be further empirically verified. On the other hand, existing research has failed to integrate individual and organizational perspectives, nor has it fully explored the contingent roles of contextual variables, such as self-efficacy and transformational leadership in the relationship between the two constructs. Specifically, will the intervention of transformational leadership alter the strength and direction of AI application's impact on employees' job insecurity? Additionally, will there be heterogeneity changes in the associative effect between AI application and employees' job insecurity when employees' self-efficacy is high?

In view of the above, this study integrates the Conservation of Resources Theory and the Cognitive Appraisal Theory of Stress to construct a theoretical model linking AI application and employees' job insecurity. Specifically, the Conservation of Resources Theory is primarily employed to explain the heterogeneous impacts of AI application on employees' job insecurity, whereas the Cognitive Appraisal Theory of Stress clarifies the moderating roles of self-efficacy and transformational leadership in this focal relationship. This study thus systematically examines the underlying mechanism through which AI application affects employees' job insecurity, as well as the possible moderating roles of self-efficacy and transformational leadership.

## 2 Theoretical analysis and hypothesis development

### 2.1 AI application and employees' job insecurity

AI, as a new generation of core technology capable of simulating and extending human intelligence, with its core capabilities such as autonomous learning, data mining analysis, and intelligent decision-making, is deeply restructuring the organizational management paradigm and work forms. Through process optimization and efficiency improvement, AI application fundamentally changes the task system, performance methods, and content structure of employees (Qin et al., 2025). In this context of intelligent transformation, employees' perception of the uncertainty in the working environment has significantly increased, and they are more likely to have concerns about the potential loss of job continuity and core job characteristics, that is, job insecurity (Brougham and Haar, 2020; Lingmont and Alexiou, 2020). The Conservation of Resources Theory posits that individuals possess an inherent motivation to acquire, maintain, and consolidate core valuable resources. Factors such as job stability and skill value fall under this category of core resources. When technological changes lead to changes in the working environment, individuals tend to anticipate potential losses of resources, which in turn gives rise to stressful psychological experiences. Job insecurity is precisely the cognitive and emotional feedback that individuals have regarding this resource threat.

In the early stage, the moderate AI application is dominated by the innovation effect, which can effectively alleviate employees' job insecurity. The application of this technology can enhance employees' work efficiency, reduce the time spent on repetitive and cumbersome tasks, and create conditions for employees to participate in complex and challenging work (Wang R. N. et al., 2025). From the perspective of Conservation of Resources Theory, this indicates that technology can generate resource gains for employees. Specifically, by reducing time waste and improving task performance quality, technology indirectly enriches employees' disposable core resources, lowers their perceived risk of resource loss, and thereby weakens the foundational conditions for job insecurity. When dealing with such work, the rational use of AI technologies can effectively increase the possibility of employees generating innovative ideas and enhance the innovative effect of AI application. On the one hand, AI application has expanded employees' access to information, reduced the costs of information acquisition and processing, enabling employees to complete basic tasks more efficiently. This allows them to allocate their limited resources such as time and energy to more creative work (Zhang et al., 2025). Such optimization of resource allocation aligns with the core logic of Conservation of Resources Theory that emphasizes proactively accumulating and efficiently utilizing resources to guard against potential losses, and further strengthens the mitigating effect of technology adoption on job insecurity. On the other hand, AI application can drive the reorganization of production factors and the optimization of work processes. It can also assist employees in handling complex tasks, stimulate their innovative thinking, thereby enhancing their professional competence and perception of work value. The improvement of professional competence itself constitutes an enhancement of core resources. According to Conservation of Resources Theory, the richer an individual's core resources, the weaker their concern about resource loss, and job insecurity naturally decreases accordingly. Furthermore, AI application helps to break the boundaries of time and space, supports remote collaboration and flexible work patterns. It also enhances the autonomy and flexibility of employees' work, and helps to meet their diverse needs, thereby increasing their sense of job control and job satisfaction (Sun et al., 2025).

However, excessive deployment of AI may trigger a substitution effect, which instead exacerbates employees' sense of job insecurity. Firstly, there is the pressures of skill iteration and job replacement. The automation of programmatic and repetitive tasks by AI not only directly replaces some job positions, but also continuously drives the structural upgrading of skill requirements. Employees are deeply trapped in the predicament of career transformation and unemployment risks due to their concerns about the depreciation of their existing skills (Zhao, 2025). Their core professional resources are directly threatened. Conservation of Resources Theory emphasizes that actual or potential resource loss serves as the core trigger of stress and negative psychological experiences. As employees' most critical occupational resources, skills and positions face substitution risks that directly elicit intense anxiety over resource loss, thereby exacerbating job insecurity. Secondly, technological anxiety and the erosion of professional identity are significant issues. The rapid iteration of AI technologies can cause some employees with insufficiently adapted skills to experience technological anxiety. Excessive reliance on AI can reinforce employees' perception that their work value is being weakened or negated. This negative evaluation will erode their self-concept and professional identity, thereby intensifying their sense of job insecurity (Kim and Kim, 2024). As an important psychological resource for individuals in the workplace, professional identity depletion falls within the scope of non-material resource loss in Conservation of Resources Theory. Such loss can further trigger individuals' concerns about their resource stock, resulting in a superimposed effect of job insecurity. Furthermore, the defensive resource conservation behavior is triggered. When employees perceive high complexity and uncertainty brought about by the excessive use of AI, and lack effective countermeasures, they will activate defensive resource conservation motivation and adopt passive psychological defense strategies to preserve the existing resources. Such reactions are usually accompanied by a higher level of job insecurity and a tendency toward behavioral withdrawal (Umair et al., 2023). This process is fully consistent with the “loss spiral” logic of Conservation of Resources Theory. When individuals perceive resource threats and lack avenues for resource replenishment, they adopt defensive behaviors such as withdrawal to reduce further resource consumption, yet such behaviors in turn intensify fear of resource loss and ultimately reinforce job insecurity.

In conclusion, the relationship between AI application and employees' job insecurity is not a simple linear one. In the initial phase of application, AI mainly exerts an innovation effect through the resource gain mechanism, effectively alleviating employees' job insecurity. However, once the application degree is overly deepened, the substitution effect dominated by the resource threat mechanism will prevail, thereby intensifying employees' job insecurity. Based on this, this study proposes Hypothesis H1.

H1: There is a positive U-shaped relationship between AI application and employees' job insecurity. Moderate AI application can reduce employees' job insecurity, while excessive AI application will increase it.

### 2.2 The moderating role of self-efficacy

Self-efficacy refers to the belief of an individual that they can successfully complete a specific task or cope with a particular situation. It reflects individuals' perceptions of their own agency and directly influences their motivation level, behavioral choices, and emotional responses when facing challenges (Bandura, 1977). Under the theoretical framework of the Cognitive Appraisal Theory of Stress, self-efficacy, as a core psychological resource, can shape the evaluation process of stressors by the individual, thereby influencing the choice of coping strategies and the final emotional outcome. This theory emphasizes that stress generation is a dynamic process of interaction between an individual and the environment, with the core lying in the “primary evaluation” and “secondary evaluation” of the situation by the individual. Primary evaluation focuses on determining the nature of events, that is, distinguishing whether the event is a threat, a challenge or a damage. In contrast, secondary evaluation assesses based on individuals' own coping resources and capabilities. In the context of AI application, employees may view it as a “challenge” to enhance efficiency, or as a “threat” to job stability. Self-efficacy is the key personal factor that influences this cognitive evaluation tendency (Han et al., 2025).

Specifically, when confronted with job changes brought about by AI application, employees with high self-efficacy are more inclined to appraise technological iterations as “challenges” that can be addressed rather than as “threats” that are uncontrollable. This cognitive tendency is fully consistent with the logic in the Cognitive Appraisal Theory of Stress that secondary appraisal outcomes dominate primary appraisal tendencies. That is, employees with high self-efficacy confirm sufficient coping resources through secondary appraisal, and thus assign positive attributes to technological change in primary appraisal. These employees have confidence in their ability to learn and adapt to new technologies. This positive secondary evaluation makes them more likely to perceive AI as an enabling tool rather than a threat of replacement (Khan et al., 2025). According to the Conservation of Resources Theory, high self-efficacy itself is an important psychological resource that can enhance employees' confidence in obtaining and preserving resources (Kong et al., 2025). Therefore, in the same context of AI application, employees with high self-efficacy perceive lower risks of resource loss and their level of job insecurity is also more likely to remain at a relatively lower level.

On the contrary, employees with low self-efficacy are more prone to forming a primary evaluation of AI application as “threats.” These employees lack confidence in their ability to cope with technological changes and acquire new skills, and they judge themselves to be deficient in resources to deal with the situation in their secondary evaluations. The transactional model of stress and coping proposes that when secondary appraisal determines that individuals lack sufficient coping resources, primary appraisal will tend to categorize events as threats first and foremost. This appraisal pathway directly amplifies insecurity among employees with low self-efficacy. This negative cognitive evaluation will amplify their anxieties about future uncertainties and personal value, thereby exacerbating the anticipated resource loss that may be caused by AI application. At this point, even if the level of AI application is moderate, employees with low self-efficacy may still experience a high level of job insecurity and may adopt defensive strategies such as avoidance and resistance, further depleting their psychological resources. From the perspective of coping strategy logic in the Cognitive Appraisal Theory of Stress, such defensive strategies represent typical individual responses to threat-oriented appraisal outcomes, while the ineffectiveness of these strategies in turn reinforces the initial negative appraisal, forming a cyclical, superimposed pattern of insecurity.

Therefore, self-efficacy, as a core personal resource and cognitive filter, can moderate the intensity of the impact exerted by AI application on employees' job insecurity. High self-efficacy can enhance the alleviating effect of moderate AI application on employees' job insecurity, while weakening the aggravating effect of excessive AI application on job insecurity. Based on this, this study proposes Hypothesis H2.

H2: Self-efficacy negatively moderates the positive U-shaped relationship between AI application and employees' job insecurity. That is, self-efficacy can strengthen the negative impact of AI application on employees' job insecurity and weaken the positive impact on employees' job insecurity.

### 2.3 The moderating role of transformational leadership

Transformational leadership refers to a leadership style characterized by charisma and inspiration, intellectual stimulation, and personalized consideration. Though these core behaviors, leaders inspire employees' higher-level needs and intrinsic motivations, thereby motivating them to transcend short-term interests and pursue collective goals and self-improvement (Franken et al., 2023). This leadership style focuses on arousing employees' pursuit of meaning, growth, and achievement, helping them make the transition from “ordinary selves” to “optimal selves.” In turn, it enhances their work performance, environmental adaptability and innovative behaviors (Jeong, 2025). In the context of work changes brought about by AI application, transformational leadership, as a critical organizational contextual variable and resource carrier, can provide employees with essential psychological and social support. It thus may potentially alter the path through which AI application affects employees' job insecurity (Wang H. Y. et al., 2025). From the perspective of the Cognitive Appraisal Theory of Stress, the support provided by transformational leadership essentially optimizes employees' conditions for secondary appraisal by supplementing external resources, reducing their concerns about insufficient coping ability, and thereby reshaping their cognitive tendency toward AI as a stressor. Furthermore, the conservation of resources theory posits that individuals have an inherent tendency to acquire, maintain, and protect valuable resources. Transformational leadership provides psychological support, empowerment, and development opportunities for employees, thereby replenishing the crucial resources need to cope with technological changes and mitigating the threat of resource depletion. Meanwhile, the social exchange theory emphasizes that high-quality leader-member exchange relationships can stimulate employees' willingness to reciprocate and their perception of trust. Transformational leadership, through personalized consideration and intellectual stimulation, establishes a stable social exchange relationship with employees. This encourages employees to view leadership support as a long-term investment when facing the uncertainties brought by AI, thereby weakening defensive mentality and alleviating job insecurity.

Specifically, high-level transformational leadership can enhance the mitigating effect of moderate AI application on job insecurity. Firstly, through depicting an attractive organizational vision aligned with AI-driven transformation, transformational leadership can help employees understand the significance and personal value of the change. This reframes uncertainty as a common development opportunity, thereby reducing their sense of confusion and threat perception about the future. This sense of charm instills hope and a sense of purpose, serving as an important psychological resource replenishment (Yu and Xiang, 2025). This process aligns with the logic of primary appraisal in the Cognitive Appraisal Theory of Stress. Through visionary guidance, leaders directly influence employees' judgment of the nature of technological change, facilitating their shift from threat-oriented appraisal to challenge-oriented appraisal. Secondly, the personalized consideration and support provided by transformational leadership can directly address employees' psychological safety needs during the period of change. Leaders' behaviors of listening, understanding and supporting enhance employees' sense of belonging and trust, enabling them to express their concerns and seek assistance, thereby alleviating the isolation and anxiety stemming from skill-related worries or role ambiguity (Ma et al., 2020). Furthermore, transformational leadership stimulates intelligence among employees, encouraging them to break free from their cognitive frameworks, acquire new skills, and explore innovative approaches to AI application. This not only directly enhances employees' capabilities to cope with technological changes, but also strengthens their sense of work control and self-efficacy through empowerment and recognition. Consequently, employees are more likely to perceive AI application as a “challenge” for skill improvement rather than a “threat” to their career survival (Czura et al., 2026).

On the contrary, in contexts characterized by low-level transformational leadership, employees often have difficulty obtaining effective supply of the aforementioned psychological and instrumental resources. Leaders often fail to provide a clear direction for change, neglect individual needs and concerns of their employees, and are also unable to stimulate employees' enthusiasm for learning and motivation for innovation. In this situation, employees need to deal with various uncertainties brought about by AI application on their own, and make them more prone to view AI as an unmanageable “threat.” According to the Cognitive Appraisal Theory of Stress, when employees lack external resource support, their secondary appraisal will judge coping resources as insufficient, thereby strengthening the threat perception of technological change in primary appraisal and forming a negative cognitive cycle. The concerns over skills depreciation and role obsolescence caused by technological changes cannot be effectively alleviated or supported at the leadership level. The perception of resource loss will be further magnified, ultimately leading to an increase in the level of job insecurity.

Therefore, transformational leadership, as a crucial external situational resource, can moderate the positive U-shaped relationship between AI application and employees' job insecurity. High-level transformational leadership can amplify the negative impact of moderate AI application on employees' job insecurity, while weakening the aggravating effect of excessive AI application on job insecurity. Based on this, this study proposes Hypothesis H3.

H3: Transformational leadership negatively moderates the positive U-shaped effect of AI application on employees' job insecurity. That is, transformational leadership can strengthen the negative impact of AI application on employees' job insecurity and weaken the positive impact on employees' job insecurity.

**Figure 1:**
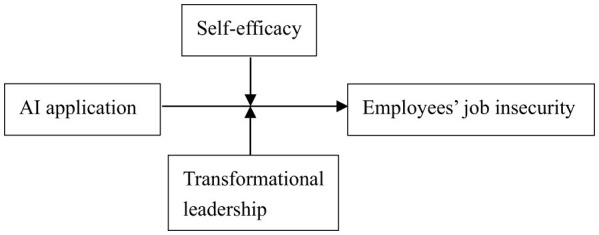
Theoretical model.

The theoretical model of this study is shown in Figure 1.

## 3 Methodology

### 3.1 Sample and data collection

This study collected primary data via a questionnaire survey to empirically test the proposed theoretical hypotheses. The questionnaire was distributed to employees of enterprises in provinces including Shandong, Hebei, Henan, Hubei, Hunan, Jiangsu, Zhejiang, and Guangdong. The samples covered multiple industries such as manufacturing, wholesale and retail, software and information technology services, and financial services. Pre-survey results showed that all the aforementioned industries are in a stage of rapid transformation and development. Employees in these industries generally experience a relatively high level of career insecurity. Meanwhile, these industries have strong demands for employees' creative performance. Based on this, the sample group selected in this study has good representativeness for exploring the relationship between AI application and employees' job insecurity. Prior to the formal distribution of the questionnaire, the research team clearly informed all respondents of the survey's anonymous nature, data confidentiality principles, and the purpose that the data would be used exclusively for academic research. These measures were taken to ensure the accuracy and standardization of questionnaire responses.

Data were collected using a mixed online and offline questionnaire distribution procedure. All measures were administered as employee self-reported questionnaires. For the online survey, an electronic questionnaire was created on the Credamo platform, which generated a unique QR code and survey link. Participants were recruited through snowball sampling: initial respondents were in-service employees from the target industries and were asked to forward the questionnaire to eligible colleagues or professional contacts. For the offline survey, paper questionnaires were distributed with the assistance of corporate human resources departments. Eligible employees were invited to complete the questionnaire on-site during an arranged time period, after which the completed questionnaires were collected immediately. Both online and offline respondents participated voluntarily and anonymously, and only questionnaires from currently employed participants in the relevant industries were retained for analysis. The survey was conducted from August to September 2025. A total of 453 questionnaires were distributed, including 242 via the online channel and 211 via the offline channel. A total of 449 questionnaires were recovered, with 242 from the online channel and 207 from the offline channel. The research team implemented strict validity screening on the recovered questionnaires. Unqualified ones were excluded, such as those completed in less than 60 s, those with significant logical contradictions in answers, and those with excessively high missing rates for key items. Finally, 411 valid questionnaires were obtained (219 online and 192 offline), resulting in an effective recovery rate of 90.73%. Analysis revealed no significant difference in the sample data collected through online and offline channels regarding AI application (*t* = 0.596, *p* = 0.507). The same non-significant results were obtained for all other variables in the analysis. This indicates that the questionnaires collected online and offline can be merged into a single sample for subsequent analyses.

### 3.2 Measures

To ensure the reliability and validity of the research measurement tools, this study selected mature scales that have been repeatedly validated in empirical studies in relevant fields at home and abroad. All involved scales are sourced from international authoritative academic journals. They have also been tested and applied in the Chinese local context, demonstrating good reliability, validity and adaptability. In the process of introducing the scales, the standardized “translation-back-translation” procedure was strictly followed. Bilingual experts were invited to participate in the review to ensure the semantic accuracy and cross-cultural equivalence of the scale items. In addition, a small-scale pre-survey was conducted before the formal research implementation. Based on the feedback from the pre-survey, the questionnaire expressions were systematically revised and optimized to further improve the clarity and applicability of the scale items. Except for demographic variables, all scales for core variables adopted a 5-point Likert scale, with 1 representing “strongly disagree” and 5 representing “strongly agree.” Respondents answered independently according to their actual situations.

#### 3.2.1 Artificial intelligence application (AIA)

The measurement scale for the variable of AI application in this study was developed based on the framework of the computer usage scale created by Medcof (1996). It underwent localized revisions by integrating the coding and analysis results of in-depth interview data from AI enterprises. Eventually, a measurement scale consisting of 3 items was formed. Representative items include “The company you work for has introduced a large number of AI technologies and related equipment” and “Core links in your work, such as reasoning, decision-making, and problem-solving, are mainly completed independently by intelligent devices.”

#### 3.2.2 Employees' job insecurity (EJI)

Drawing on the measurement scale of job insecurity developed by Hellgren et al. (1999), this study adjusted the item “I feel that [the organization] can provide me with a stimulating job content in the near future” to “You think the organization will increase the challenge of your work in the future” in line with the research context to measure the variable of employees' job insecurity, while retaining all other original items. This scale consists of 7 measurement items, such as “You feel anxious about the possibility of losing your job in the future”, “You think the organization will increase the challenge of your work in the future”, and “You believe the organization will require you to have stronger work abilities in the future.”

#### 3.2.3 Self-efficacy (SE)

To measure the variable of self-efficacy, this study adopted the mature scale developed by Schwarzer et al. (1995). This scale includes 6 measurement items in total. Representative items mainly include “You are very confident in your ability to effectively deal with any unexpected events”, “With your intelligence, you can cope with unforeseen work situations”, and “When facing difficulties, you can usually find multiple solutions.”

#### 3.2.4 Transformational leadership (TL)

To measure the variable of transformational leadership, this study adopted the mature scale developed by Chang and Lee (2007). This scale consists of 8 measurement items, such as “The leader often analyzes the impact of employees' work on the organization's overall goals together with them”, “The leader cares about employees' work and life, and sincerely provides suggestions for their development”, and “You believe the leader is open-minded and has a strong sense of innovation.”

#### 3.2.5 Control variables

Existing studies have confirmed that demographic characteristic variables exert a significant impact on employees' job insecurity. In view of this, when testing the relationships among core variables, this study included gender, age, education, length of service (LoS), and job type in the category of control variables. Specifically, regarding gender, this study codes “1” for males and “0” for females. For age, “1” represents “25 years old or younger”, “2” denotes “26–35 years old”, “3” stands for “36–45 years old”, “4” indicates “46–55 years old”, and “5” corresponds to “56 years old or above.” In terms of educational attainment, “1” signifies “high school or below”, “2” represents “college diploma”, “3” denotes “bachelor's degree”, “4” stands for “master's degree”, and “5” indicates “doctoral degree.” Regarding length of service, “1” is coded for “5 years or less”, “2” for “6–10 years”, “3” for “11–15 years”, “4” for “16–20 years”, and “5” for “more than 20 years.” For job type, “1” represents “technical positions” and “0” denotes “non-technical positions.” These measures aim to eliminate the interference of irrelevant variables on the causal relationships between core variables and ensure the reliability of the research conclusions.

## 4 Results

### 4.1 Descriptive statistics and correlation analysis

This study used SPSS 17.0 statistical analysis software to conduct hierarchical multiple linear regression analysis on all research variables. The sample data were centered to avoid potential multicollinearity issues. A variance inflation factor (VIF) test was performed for each variable. The results showed that the VIF _max_ was less than 10, indicating no multicollinearity among the variables. The demographic characteristics of the valid samples in this study are as follows. In terms of gender distribution, male samples accounted for 40.39% and female samples for 59.61%. Regarding educational background, 62.53% of the samples had a bachelor's degree and 20.19% had a master's or doctoral degree. Knowledge workers with a bachelor's degree or above accounted for 82.72% of the total, forming the main body of the sample. The age distribution showed a significant younger trend. 25.55% of the respondents were 25 years old or younger, 51.09% were between 26 and 35 years old, and young employees aged 35 or below accounted for as high as 76.64%. In terms of work experience, 44.28% of the samples had worked for 5 years or less, 30.90% for 6 to 10 years, and 13.38% for 11 to 15 years. Employees with 15 years or less of work experience accounted for 88.56% of the total, reflecting an overall characteristic of relatively short service tenure.

The results of the correlation analysis, as shown in Table 1, indicate a significant negative correlation between AI application and employees' job insecurity. This finding is fully consistent with the theoretical expectations and provides preliminary empirical evidence to support the research hypotheses proposed in this study.

**Table 1:** Descriptive statistical analysis.

Variables	Average	SD	1	2	3	4	5	6	7	8	9
1. Gender	–	–	1								
2. Age	–	–	0.0803	1							
3. Education	3.139	0.744	−0.0135	−0.192^***^	1						
4. LoS	2.000	1.200	0.0538	0.817^***^	−0.287^***^	1					
5. Job type	0.326	0.469	0.0304	−0.070	−0.115^**^	−0.095^*^	1				
6. AIA	3.603	0.694	0.0227	0.014	0.034	0.003	0.069	1			
7. SE	3.765	0.598	0.0026	0.079	0.048	0.007^*^	−0.105	0.364^***^	1		
8. TL	3.780	0.710	0.0187	0.052	−0.022	0.051	0.004	0.394^***^	0.517^***^	1	
9. EJI	2.794	0.5553	−0.074	−0.121^**^	−0.102^**^	−0.116^**^	0.020	−0.155^***^	−0.378^***^	−0.344^***^	1

Two-tailed test. ^***^p < 0.001, ^**^p < 0.01, ^*^p < 0.05.

### 4.2 Common method deviation test

The sample data of this study were all collected through employee self-reports, a data collection method that is prone to common method bias. In view of this, a series of procedural control measures were adopted during the research process to avoid such bias, including ensuring the anonymity of the survey and revising and improving measurement items based on pre-survey results. To further test the degree of impact of common method bias, this study used Harman's single-factor test and conducted exploratory factor analysis on all scale items. The test results showed that the first principal component factor explained only 32.30% of the total variance, which is lower than the critical criterion of 40%. Furthermore, this study employed a latent methods factor CFA to test for common method bias. The results showed that, compared with the three-factor model, the single-factor model yielded the following fit indices. The χ^2^/df was 11.362, NFI was 0.537, CFI was 0.558, TLI was 0.516, and RMSEA was 0.159. These values demonstrate a poor fit of the single-factor model, confirming that the risk of CMV in this study is within an acceptable range and does not substantially interfere with data quality or subsequent analyses. Thus, the data are suitable for hypothesis testing.

### 4.3 Reliability and validity test

The main measurement items of each core variable are presented in Table 2. Results of the reliability test show that the Cronbach's α coefficients and composite reliability (CR) of all variables are greater than 0.7, indicating that the measurement reliability of each variable meets the standard requirements and has good dependability. Results of the validity test demonstrate that the factor loading coefficients of the measurement items for each variable are basically greater than 0.7, the average variance extracted (AVE) values are greater than 0.6, and the KMO values are greater than 0.7. These findings confirm that all variables have good validity.

**Table 2:** Results of reliability and validity.

Variables	Items	Factor loadings	CR	AVE	Cronbach's α	KMO
AIA	AIA1	0.861	0.899	0.748	0.829	0.723
	AIA2	0.874				
	AIA3	0.860				
EJI	EJI1	0.793	0.929	0.651	0.910	0.895
	EJI2	0.822				
	EJI3	0.852				
	EJI4	0.799				
	EJI5	0.842				
	EJI6	0.819				
	EJI7	0.715				
SE	SE1	0.787	0.916	0.646	0.890	0.901
	SE2	0.793				
	SE3	0.776				
	SE4	0.845				
	SE5	0.824				
	SE6	0.796				
TL	TL1	0.799	0.941	0.666	0.927	0.943
	TL2	0.817				
	TL3	0.667				
	TL4	0.846				
	TL5	0.843				
	TL6	0.853				
	TL7	0.859				
	TL8	0.829				

To further examine the discriminant validity of each variable, this study employed the AMOS 17.0 to conduct a confirmatory factor analysis (CFA). As shown in the results of the confirmatory factor analysis for the four-factor model in Table 3, χ^2^/df = 1.424, which meets the goodness-of-fit criterion of 1 < χ^2^/df < 3. Other indicators are as follows, RMSEA = 0.032 (less than 0.05), NFI = 0.950, CFI = 0.984, TLI = 0.980, and PNFI = 0.750. All the above indicators demonstrate that the theoretical model of this study has a good fit with the sample data. In summary, all variables in this study meet the requirements of reliability and validity tests, and the research model exhibits good robustness.

**Table 3:** Results of confirmatory factor analysis.

Model	χ^2^/df	NFI	CFI	TLI	PNFI	RMSEA
Single-factor	11.362	0.537	0.558	0.516	0.490	0.159
Two-factor	5.982	0.761	0.792	0.767	0.681	0.110
Three-factor	4.616	0.819	0.852	0.831	0.718	0.094
Four-factor	1.424	0.950	0.984	0.980	0.750	0.032

### 4.4 Main effect test

**Figure 2:**
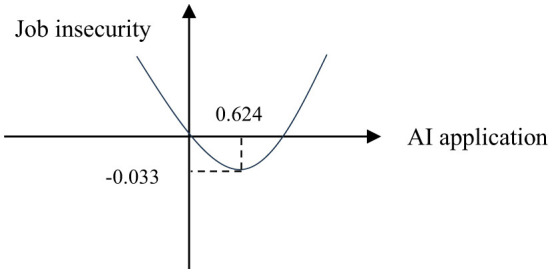
The relationship between AI application and employees' job insecurity.

A U-shaped relationship for the main effect requires the satisfaction of the following three conditions. (1) The coefficient of the squared term of the independent variable x is significantly positive; (2) the slope k of the curve is significantly negative when x takes its minimum value and significantly positive when x takes its maximum value; (3) the inflection point of the curve lies within the valid rang of *x*. First, as shown in Model 3 of Table 4, the coefficient of the linear term of AI application is significantly negative (β1 = −0.106, *p* < 0.01), and the coefficient of its squared term is significantly positive (β_2_ = 0.085, *p* < 0.05). Second, based on β1 = −0.106 and β_2_ = 0.085, the slope of the curve can be derived as *k* = −0.106 + 0.170*x*. After standardization, the value range of *x* is −3 to 3; the value of *k* is significantly negative when *x* = −3 and significantly positive when *x* = 3. Finally, the inflection point value of *x* is calculated as –β1/2β_2_ = 0.624, which falls within the valid range of *x*. These results indicate that after incorporating the control variables, there is a significant U-shaped relationship between AI application and employees' job insecurity. Moderate application of AI can significantly reduce employees' job insecurity (β = −0.120, *p* < 0.001), while excessive use of AI will significantly increase employees' job insecurity (β = 0.085, *p* < 0.01). Therefore, Hypothesis H1 is supported. The relationship between AI application and employees' job insecurity is shown in Figure 2.

**Table 4:** Results of regression analyses of AI application on employees' job insecurity.

Variables	M1	M2	M3	M4	M5	M6	M7
Constant	0.496^***^	0.483^***^	0.449^***^	0.422^***^	0.330^**^	0.505^***^	0.465^***^
Gender	−0.075	−0.072	−0.081	−0.074	−0.092^*^	−0.065	−0.084
Age	−0.032	−0.029	−0.030	−0.031	−0.035	−0.025	−0.033
Education	−0.110^***^	−0.106^***^	−0.108^***^	−0.090^***^	−0.081^**^	−0.115^***^	−0.110^***^
LoS	−0.051	−0.051	−0.050	−0.032	−0.026	−0.042	−0.042
Job type	0.029	0.041	0.0451	0.027	0.036	0.040	0.036
AIA		−0.120^***^	−0.106^***^	−0.021	0.018	−0.026	0.020
AIA^2^			0.085^**^		0.095^**^		0.040
SE				−0.315^***^	−0.280^***^		
AIA ^*^ SE				−0.127^*^			
AIA^2^^*^ SE					−0.120^*^		
TL						−0.258^***^	−0.200^***^
AIA ^*^ TL						−0.112^**^	
AIA^2^^*^ TL							−0.122^**^
R^2^	0.040	0.062	0.069	0.177	0.185	0.163	0.167
F	3.78^***^	4.99^***^	5.58^***^	10.72^***^	9.89^***^	9.30^***^	9.61^***^

Two-tailed test. ^***^p < 0.001, ^**^p < 0.01, ^*^p < 0.05.

### 4.5 Test of the moderating effect of self-efficacy

As shown in Table 4, self-efficacy negatively moderates the U-shaped relationship between AI application and employees' job insecurity. Specifically, self-efficacy can strengthen the negative impact of AI application on employees' job insecurity (β = −0.127, *p* < 0.05). This is because, according to the Conservation of Resources Theory, high self-efficacy constitutes employees' psychological resource. Such a resource can enhance employees' confidence and capability in coping with technological transformations. When enterprises apply artificial intelligence moderately, employees with high self-efficacy tend to regard this technology as a tool for improving work efficiency. They will take the initiative to leverage artificial intelligence to optimize work processes, cut down on repetitive tasks, and thereby strengthen the mitigating effect of artificial intelligence on employees' job insecurity. It can also weaken the positive impact of AI application on employees' job insecurity (β = −0.120, *p* < 0.05). When the application of artificial intelligence becomes excessive, employees with high self-efficacy are more inclined to appraise the technological impact as a “manageable challenge” rather than an “uncontrollable threat.” They will take the initiative to acquire new skills for collaborating with AI and reconstruct their own occupational value, thereby mitigating the sense of job insecurity caused by the technological substitution effect. In contrast, employees with low self-efficacy lack confidence in coping with technological transformations. Even when AI is applied moderately, they are prone to experiencing anxiety. When the deployment of such technology becomes excessive, the sense of job insecurity among these employees will be further amplified. Therefore, Hypothesis H2 is supported.

### 4.6 Test of the moderating effect of transformational leadership

As shown in Table 4, transformational leadership negatively moderates the U-shaped relationship between AI application and employees' job insecurity. Specifically, transformational leadership strengthens the negative impact of AI application on employees' job insecurity (β = −0.112, *p* < 0.01). Transformational leaders establish an external support system for employees to cope with technological transformation through behaviors such as visionary motivation, individualized consideration, and intellectual stimulation. In the phase of moderate AI application, transformational leaders clearly communicate the value of technological implementation. They help employees understand the positive significance of AI in optimizing work processes, enhance employees' acceptance of technology, and thereby amplify the effect of AI in mitigating employees' job insecurity. It also weakens the positive impact of AI application on employees' job insecurity (β = −0.122, *p* < 0.01). In the phase of excessive AI application, transformational leaders provide targeted resources such as skill training and career planning guidance. They help employees break through skill bottlenecks, rebuild occupational competitiveness, and alleviate employees' fears of technological substitution, thereby mitigating the negative impacts caused by the over-adoption of AI. In contrast, in organizations with low levels of transformational leadership, leaders often lack effective guidance and support for their employees. When confronted with AI-driven transformations, employees tend to be left in a state of isolation and helplessness. Regardless of whether the level of AI application is high or low, employees' sense of job insecurity can hardly be effectively alleviated. Therefore, Hypothesis H3 is supported.

## 5 Discussion and conclusion

### 5.1 Discussion

Firstly, based on the Conservation of Resources Theory, this study empirically verifies the U-shaped relationship between the two variables. Specifically, moderate application exerts an innovation effect through a resource gain mechanism, while excessive application triggers a substitution effect through a resource threat mechanism. This finding responds to the “automation-augmentation paradox” proposed by Raisch and Krakowski (2021). It explains the differentiated impacts of AI application intensity on employees' psychological perceptions, and enriches the theoretical system of the relationship between technological change and employees' psychology. Extant literatures have not reached a consistent conclusion on the impact of AI application. Some studies focus on its substitution effect, arguing that technological application directly threatens employees' occupational resources and intensifies job insecurity (Yam et al., 2023; Zhao, 2025). Other studies emphasize its innovation and empowerment effect, claiming that technology alleviates employees' stress by optimizing work processes and improving efficiency (Li et al., 2024; Yang et al., 2025). The heterogeneous effects on employees' job insecurity possibly caused by differences in application intensity have rarely been mentioned.

Secondly, this study introduces self-efficacy (as an individual psychological resource) and transformational leadership (as an organizational situational factor) as moderating variables. It verifies that both variables negatively moderate the aforementioned U-shaped relationship. They not only strengthen the alleviating effect of moderate AI application but also weaken the exacerbating effect of excessive application. This result extends the research conclusion of Han et al. (2025) regarding “the influence of cognitive appraisal on technology adaptation.” It also echoes Yuan's (2025) demonstration of the buffering effect of transformational leadership. It clarifies the contingency conditions under which AI application affects employees' job insecurity and enriches the research on multi-level boundary mechanisms in this field. Relevant studies mostly explore the roles of individual or organizational variables separately. For instance, Kim and Lee (2025) only focus on the individual driving effect of self-efficacy on technology adaptation. Liu and Nie (2024) emphasize the independent impact of transformational leadership on employee innovation. However, neither of these studies has incorporated both types of variables into the relational framework linking AI application and employees' job insecurity.

Furthermore, this study innovatively integrates the Conservation of Resources Theory and Cognitive Appraisal Theory. For example, this study primarily employs the Conservation of Resources Theory to explain the main effect mechanism through which AI application affects job insecurity via the dual paths of resource gain and resource threat. Additionally, this study draws on the Cognitive Appraisal Theory of Stress to elucidate the functional logic underlying the moderating effects of self-efficacy and transformational leadership. Existing studies mostly rely on either the Conservation of Resources Theory or the Cognitive Appraisal Theory of Stress alone to analyze the relationship between technology and employees' psychology (e.g., Peltokorpi and Allen, 2024; Jeong and Jeong, 2025). They lack systematic interpretations based on cross-theoretical integration. This cross-theoretical integration of this study not only responds to the theoretical viewpoint proposed by Schwarzer et al. (1995) that self-efficacy serves as a core psychological resource. It also expands the application scenarios of the Conservation of Resources Theory in the context of technological change. It provides a new cross-theoretical integration framework for subsequent studies to analyze the complex correlation between technology and employees' psychology.

### 5.2 Conclusion

Based on the Conservation of Resources Theory and the Cognitive Appraisal Theory of Stress, this study explores the mechanism underlying the impact of AI application on employees' job insecurity. It also tests the moderating effects of self-efficacy and transformational leadership on the relationship between the two variables. Through empirical analysis of data from 411 valid questionnaires, this study draws the following core conclusions. First, there is a significant U-shaped relationship between AI application and employees' job insecurity. Specifically, moderate application of AI can effectively alleviate employees' job insecurity, while excessive application will intensify employees' perception of job insecurity. Second, self-efficacy plays a negative moderating role in the U-shaped relationship between AI application and employees' job insecurity. To be specific, self-efficacy can strengthen the negative alleviating effect of moderate AI application on employees' job insecurity. At the same time, it can weaken the positive intensifying effect of excessive AI application on employees' job insecurity. Third, transformational leadership also exerts a negative moderating effect on the U-shaped relationship between AI application and employees' job insecurity. In other words, transformational leadership can enhance the negative inhibiting effect of moderate AI application on employees' job insecurity. It can also reduce the positive promoting effect of excessive AI application on employees' job insecurity.

### 5.3 Managerial implications

First, organizations should fundamentally alleviate employees' job insecurity to activate their work efficiency and innovation potential. On the one hand, managers need to establish a transparent communication mechanism for job-related decisions, strengthen job stability guarantees, and build psychological security through emotional support and positive incentives. Meanwhile, supporting professional psychological counseling services should be provided to guide employees to perceive the value of AI application with a positive attitude, clarify the enabling effect of technology on both organizations and individuals, and resolve negative emotions and anxiety. On the other hand, targeted knowledge and skills upgrading training should be carried out, focusing on the capabilities required for high-difficulty and high-value tasks. These programs can help employees adapt to the upgraded job requirements driven by AI. This not only stimulates work initiative through a sense of value and achievement, but also reduces insecurity through enhanced capacities.

Secondly, organizations need to view the dual impacts of AI rationally and avoid overemphasizing a single effect. They should not only prevent the amplification of job insecurity caused by technological substitution, but also guard against employee slackness and creativity reduction resulting from excessive empowerment by technology. Based on the U-shaped relationship conclusion of this study, organizations should precisely control the intensity of AI application. They should focus it on process optimization and the reduction of basic tasks, thereby creating space for employees to engage in creative work. At the same time, regular specialized training on AI application is conducted to help employees deepen their understanding of the technical value, master practical operation methods, and guide them to regard technology as a tool for improving efficiency rather than a substitution threat. By stimulating employees' creative behaviors in responding to challenges, long-term benefits such as stable employment and personal development can be achieved.

Thirdly, self-efficacy, as the critical psychological resource for buffering the impact of technological changes, should be incorporated into the priorities of organizational human resource management. Organizations can conduct specialized psychological training to enhance employees' confidence and ability to cope with AI-driven changes. These measures can guide employees to proactively adapt to changes in the working environment with an optimistic and positive mindset. This will reduce their sense of job insecurity from an internal perspective, and simultaneously improve their job competence and risk resistance capabilities.

In addition, organizations should strive to create a positive and inclusive atmosphere for change. The core of this effort lies in cultivating a team of transformational leaders and establishing smooth communication mechanisms between superiors and subordinates. Transformational leaders need to strengthen employees' sense of organizational belonging through vision inspiration, individualized care and intellectual stimulation. Meanwhile, they should establish transparent information transmission channels to respond timely to employees' concerns about technological application and career development, thereby resolving the anxiety caused by uncertainty. Through the dual guarantees of leadership empowerment and environmental support, organizations can weaken the negative impact of job insecurity and stimulate employees' initiative and creativity in participating in organizational change.

### 5.4 Limitations and future directions

Firstly, although the sample of this study covers multiple industries such as manufacturing and finance, it does not conduct a stratified analysis based on industry technology intensity (e.g., high-tech industries and traditional industries) and occupational skill attributes (e.g., technical, clerical, and creative occupations). There are differences in the depth and scenarios of AI application across different industries, and there are essential distinctions in the skill substitutability of different occupations. These factors may lead to heterogeneous effects of AI application on employees' job insecurity, which have not been explored in this study and thus limit the generalizability of the conclusions to a certain extent. Future research can conduct stratified sampling according to the technological attributes of industries and the types of occupational skills. It can further explore the differentiated mechanisms underlying the impact of AI application on employees' job insecurity across different groups, so as to improve the pertinence and generalizability of research conclusions.

Secondly, this study focuses on the mechanism of individual-level variables, without incorporating key contextual variables at the organizational and industrial levels. External factors such as organizational culture (e.g., innovation-inclusive culture and conservative culture), organizational support (e.g., training support and psychological support), and industrial competition intensity may all influence the relationship between AI application and employees' job insecurity by moderating the process of resource acquisition or stress perception. Their potential mechanism of action has not yet been explored. Future research can incorporate organizational and industrial-level variables such as organizational culture, perceived organizational support, and industrial competitive environment. It can test their moderating or mediating effects on the relationship between AI application and employees' job insecurity, so as to enrich the theoretical system in this field.

In addition, this study adopted a cross-sectional questionnaire survey method to collect data. It can only verify the correlation and moderating effects between variables, but cannot reveal the dynamic causal relationship between AI application and employees' job insecurity. With the iterative upgrading of AI technology and the advancement of employees' adaptation process, the relationship between the two may show phased changes, which cannot be captured by static data. Future research can conduct longitudinal follow-up surveys or cross-time comparative analyses. It can track the changes in the intensity of AI application over a long period, while paying attention to the dynamic fluctuations of employees' job insecurity, so as to clarify the long-term evolution law of the relationship between the two variables.

## Data Availability

The original contributions presented in the study are included in the article/supplementary material, further inquiries can be directed to the corresponding author.
